# Paroxysmal Non-Kinesigenic Choreoathetosis Case Report and a Review of the Pathogenesis

**DOI:** 10.7759/cureus.21804

**Published:** 2022-02-01

**Authors:** Ramiz H Kara, Gyusik Park, Shoeb B Lallani, Hassan N Kesserwani

**Affiliations:** 1 Neurology, University of North Texas Health Science Center, Texas College of Osteopathic Medicine, Fort Worth, USA; 2 Neurology, University of Alabama at Birmingham School of Medicine, Birmingham, USA; 3 Neurology, Flowers Medical Group, Dothan, USA

**Keywords:** dystonia, athetosis, chorea, paroxysmal non-kinesigenic dyskinesia, paroxysmal kinesigenic dyskinesia, dyskinesia, movement disorders

## Abstract

Paroxysmal dyskinesias are a rare group of episodic movement disorders characterized by any combination of dystonia, chorea, and athetosis. Patients usually present early in life with episodes of variable frequency involving the limbs or facial muscles that can be disabling. In this article, we present a case of paroxysmal non-kinesigenic dyskinesia that was responsive to the sodium-channel blocker carbamazepine. Recent data has revealed the role of voltage-gated sodium channels in the pathophysiology of the disease; hence, these disorders are referred to as channelopathies. Further advancements in genetic analysis have elucidated targets corresponding to these disorders, indicating a possible role for gene sequencing in helping to differentiate the subtypes of paroxysmal dyskinesias. This case report sheds light on the pathophysiology of the various channelopathies, especially the findings of cerebellar spreading depolarization and its implication in paroxysmal kinesigenic and non-kinesigenic dyskinesias.

## Introduction

Paroxysmal dyskinesias (PD) represent a rare group of movement disorders characterized by episodic, abnormal, involuntary movements typically of the limbs. They are often provoked by a trigger and can present with any combination of dystonia, chorea, and athetosis. PD most often presents earlier in life and tends to decrease in frequency with age. Clinically, it can be difficult to differentiate PD from other forms of paroxysmal movement disorders; therefore, a careful and nuanced clinical description of the semiology of the disorder is important in making the diagnosis. Further, the differential diagnosis can span beyond movement disorders to include simple partial seizures, transient ischemic attacks, motor tics, and functional neurological disorders. Determining provoking triggers, duration, evolution and resolution, frequency, and stereotypy are essential to history taking and to help characterize this group of disorders.

The operational definition of dystonia is stereotypic, excessive hyperactivity and co-contraction of agonist-antagonist muscles across a joint leading to phasic oscillations or static posturing that can be painful and interfere with the motion and mechanics of a joint [1]. Unlike dystonia, chorea is described as an irregular, randomly-presenting sequence of involuntary jerk-like movements [2]. Athetosis is characterized by continuous, involuntary writhing movements and rarely presents in isolation, as it is usually accompanying chorea and dystonia. [2]. To date, the differentiation of paroxysmal kinesigenic dyskinesia (PKD) and paroxysmal non-kinesigenic dyskinesia (PNKD) is based upon the semiology, which includes whether an attack is motion-induced or spontaneous and the duration of an attack, with PKD episodes lasting seconds to minutes and PNKD episodes lasting minutes to hours. Therapy is empiric and may include sodium channel blockers, such as carbamazepine, or carbonic anhydrase inhibitors, such as acetazolamide. These treatment options are known to work for other paroxysmal movement disorders as well. However, with the advancement of genotyping and identification of genetic mutations of various ion-channel genes, the definition of these disorders is now further refined, and genotype-phenotype correlations are expanding rapidly [3].

## Case presentation

We present a 33-year-old male with no significant past medical history who presented to the clinic with a two-day history of dystonic posturing of his left hand and arm. These consisted of disabling bending of the elbow with flexion and posturing of the fingers with no oculo-bulbar or sensory symptoms. The spells occurred at random intervals throughout the day and were not initially associated with a known trigger, but later on, he discovered that caffeine consumption provoked the episodes. The episodes usually lasted several minutes, but one episode lasted nine hours. The spells can be painful with the inability to volitionally relax the afflicted arm and hand. Mental lucidity was maintained throughout and after the episode without a post-ictal phenomenon, and the spells disappeared during sleep. They initially began in his left arm but later spread to the right arm, occurring independently in both arms at random intervals throughout the day. The patient denied any family history of movement disorders. Socially, he abstained from smoking and alcohol, and he was not taking any medications.

On examination, his blood pressure was 129/79 mmHg with a heart rate of 77 beats per minute. His weight was 195 pounds (88.5 kilograms) with a height of six-foot (182.9 centimeters), yielding a body-mass index of 26.4. His conversations were lively with normal prosody and without dysarthria. Gait was of the normal base, stride, and cadence with normal tandem-, heel-, and toe-walking. Cranial nerve examination was entirely normal, with ocular motion intact in all directions without nystagmus. Facial power was symmetric and full. There was an absence of oro-buccolingual dyskinesias. Careful examination of the cervical musculature did not reveal any evidence of cervical dystonia. Examination of the oropharynx revealed a brisk gag reflex without palatal myoclonus.

Power testing of both upper and lower extremities was graded 5/5 in all major muscle groups with the medical research council scale. Deep tendon reflexes were 2+ and symmetric in the arms and legs. Spasticity was absent in the arms and legs along with absent Hoffman and finger flexor reflexes bilaterally. No cogwheeling was detected at the wrist with the Froment maneuver. The Babinski sign was absent with bilateral plantar strokes. Sequence motion of the fingers of both hands, finger-to-nose, and heel-to-shin maneuvers were accurate and symmetric bilaterally.

**Figure 1:**
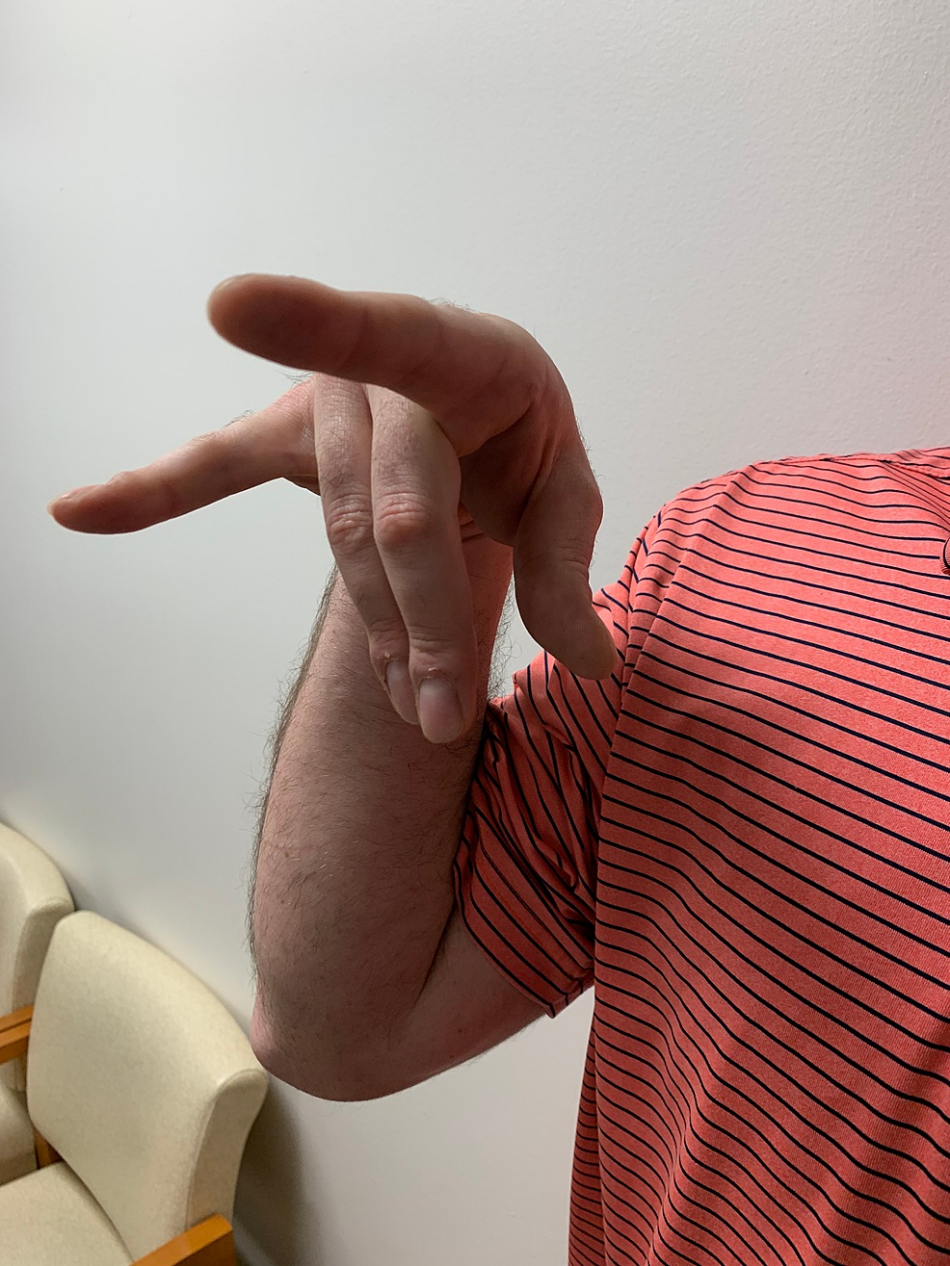
Dystonic posturing of the right arm demonstrating a 90-degree flexion at the elbow with hyperflexion of the middle two fingers at the metacarpophalangeal joints with the hand demonstrating a bovine longhorn posture

During his clinic visit, we were able to capture one of the episodes. Dystonic posturing began with a 90-degree flexion of his left elbow with radiation down to the ipsilateral hand. The resulting posture consisted of a flexed elbow joint with flexion of the hand at the first, third, and fourth fingers at the metacarpophalangeal joints with extension of the proximal and distal interphalangeal joints; the second and fifth fingers remained extended, giving the hand the appearance of a bovine longhorn (Figure 1).

An electroencephalogram (EEG) did not reveal any focal epileptiform discharge. A magnetic resonance imaging (MRI) study of the brain with and without contrast enhancement was normal. It is important to note that a T1-weighted image did not reveal any metal deposition in the basal ganglia. Fluid-attenuated inversion recovery sequences did not reveal any white matter disease. Gradient-echo and susceptibility-weighted images did not reveal any hemosiderin deposits in the basal ganglia. Serum chemistries, including free and total calcium and a thyroid screen, were normal. A comprehensive paraneoplastic panel and an antiphospholipid antibody screen were also negative. Given the semiology of symptoms and negative neuroimaging and laboratory workup, a diagnosis of paroxysmal non-kinesigenic choreoathetosis was given. Due to the risk of dependence and tolerance with benzodiazepines, he was prescribed carbamazepine, a dose of 200 milligrams (mg) twice a day, which resolved his symptoms, and he reported no further episodes on subsequent clinic visits. A functional movement disorder, a reasonable differential diagnosis, was ruled out as the patient had no psychiatric history and responded exquisitely to carbamazepine without requiring psychological counseling. 

## Discussion

PD is a rare class of movement disorders characterized by recurrent, intermittent periods of abnormal involuntary movements consisting of a combination of dystonia, chorea, and athetosis. The current classification system of paroxysmal movement disorders (PMD) is based solely on the precipitating factors of the attack with separation into four major classifications: PKD, PNKD, paroxysmal exertion-induced dyskinesia (PED), and paroxysmal hypnogenic dyskinesia (PHD). Genetic causes can be primary (familial or idiopathic) or secondary [4].

The clinical symptomatology of all four subtypes of PD varies slightly but can be useful in distinguishing between them. In PKD, symptoms often are preceded by sudden movement, resulting in dystonic posturing of one or more extremities [5-7]. Consciousness is intact, and the duration of dystonic posturing can last from seconds to minutes. However, these episodes are known to occur many times throughout the day, with some patients reporting upwards of 100 attacks a day. In PNKD, attacks are preceded by caffeine, alcohol, fatigue, stress, which can lead to episodes of dystonia, athetosis, or chorea starting from one limb and gradually spreading to other limbs and facial musculature without alterations in consciousness. Symptoms tend to last from minutes to hours but have variable frequency, occurring multiple times a week or simply a few episodes in a lifetime [8-10]. In PED, attacks are preceded by prolonged exercise. Symptoms present as leg dystonia ranging in duration from a couple of minutes to a couple of hours with daily or monthly occurrence. In PHD, symptoms present with attacks of ballism of the limbs during initiation of non-rapid eye movement sleep that results in sudden awakening, dystonia, and chorea. Duration ranges from 30 seconds to under an hour and can occur multiple times a night or a few times throughout the year. With all PD, symptoms tend to begin early in life and decrease in frequency with age [11-13]. Given our patient’s caffeine-induced dystonic posturing and responsiveness to carbamazepine, we classified his clinical presentation as PNKD.

The diagnosis of PD can be nuanced and challenging as it requires a detailed account of the clinical presentation, careful attention to past medical history, analysis of precipitating factors, and frequency and duration of episodes. An EEG should be done to investigate a possible underlying epileptic disorder, and conditions such as epilepsia partialis continua should be considered. Furthermore, an MRI brain with and without contrast enhancement should be performed to rule out focal lesions of the basal ganglia. Laboratory workup should include a metabolic profile and should include serum calcium, both ionized and total. A detailed medication profile should also be conducted to elucidate possible secondary causes of PD, such as dopamine antagonists. PD is considered a diagnosis of exclusion, and a strong clinical suspicion for the disorder helps arrive at a timely diagnosis and obviates the need for an expensive work-up. Although these qualitative measures can be used to make a presumptive diagnosis of PD, further research in the field is needed to improve the diagnostic criteria for this range of disorders.

Recent advances in the genomic analysis have resulted in the identification of many genes involved in the pathology of PD and its subtypes. The most notable genes implicated in PD include proline-rich transmembrane protein 2 (PRRT2), PNKD, TBC1 domain family member 24 (TBC1D24), and solute carrier family 2 member 1 (SLC2A1). The PRRT2 mutation is an autosomal dominant mutation implicated in the PKD subtype and less commonly in PNKD that leads to a loss-of-function change in neuron-specific protein located at the pre-synaptic level, causing interference with voltage-dependent sodium channels and soluble N-ethylmaleimide-sensitive factor (NSF) attachment receptor (SNARE) complex assembly [14]. PNKD, originally called myofibrillogenesis regulator-1 (MR-1), encodes for presynaptic terminal proteins involved in inhibition of neurotransmitter release, and its mutation creates a gain-of-function change leading to disruption of nigrostriatal neurotransmission [15]. TBC1D24 also encodes for a presynaptic protein involved in vesicle trafficking with its mutation largely implicated in paroxysmal episodic dyskinesia due to consequential aberrant neurotransmission [16]. In contrast to these genes, SLC2A1 encodes for glucose transporter 1 (GLUT-1), a glucose carrier highly expressed in the blood-brain barrier. Mutation of this protein results in neuroglycopenia due to insufficient glucose transport, which is largely implicated in the pathophysiology of PD and episodic ataxia.

Although the molecular pathologic process of PD has not been entirely elucidated, it is believed that most genetic mutations implicated in PD and its subtypes involve pre-synaptic proteins that regulate synaptic vesicle fusion (PRRT2, PNKD, TBC1D24) and consequently affect neurotransmission. As expected from the similar phenotype across different PD subtypes, the different genetic mutations all converge into a limited range of pathologic pathways that ultimately leads to neurotransmission disruption, mostly in the cerebellar and striatal circuits [17]. A recent study by Lu et al. further elucidates this mechanism by drawing an association between PRRT2 deficiency and increased induction of cerebellar spreading depolarization (SD), demonstrating that SD can cause a depolarization block of Purkinje cells and disturb cerebellar outputs, leading to dyskinetic movements in PKD [18]. The study also confirmed the effect of the PRRT2 mutation on the SNARE complex and the voltage-gated sodium channels. With further experimentation, the team was able to find that the inactivation of sodium channels was sufficient to block cerebellar SD. This result may help to explain the effectiveness of carbamazepine in the treatment of PKD [18]. The effectiveness of carbamazepine in treating the symptoms of our PNKD patient may suggest that similar pathophysiology may be implicated between PNKD and PKD, although future research should focus on elucidating the role of SD in the pathologic process of the various PD subtypes.

## Conclusions

PNKD is a rare movement disorder characterized by focal paroxysmal dystonic episodes, lasting minutes to hours, of variable frequency and usually triggered by caffeine, alcohol, stress, or periods of high emotion. Lying on the spectrum of PD, PNKD is not provoked by sudden movement and does not involve repetitive or rhythmic movements but rather sustained muscle contractions. Recent data shows that the pathophysiology of all PD subtypes similarly involves dysregulation of pre-synaptic neurotransmission. Blocking of voltage-gated sodium channels has shown to be effective for PD in experimental settings, and clinical symptoms in our patient were successfully treated with the sodium channel blocker carbamazepine. Although not currently widely available, further research may elucidate a role for genetic testing in the diagnosis of PD subtypes. Furthermore, research into the pathophysiology of the various subtypes of PD may help to elucidate alternative therapies to treat this group of disorders.
